# Energy drink-induced acute kidney injury: a case report and review of the literature

**DOI:** 10.1186/s13256-025-05614-3

**Published:** 2025-10-21

**Authors:** Ahmet Murt

**Affiliations:** 1 Cerrahpasa Medical Faculty, Department of Nephrology, Istanbul University-Cerrahpasa, Istanbul, Turkey; 2 Department of Nephrology, Bingol State Hospital, Bingol, Turkey

**Keywords:** Energy drink, Acute kidney injury, AKI, ATN, Case report

## Abstract

**Background:**

Nephrotoxic insults are among the most common causes of acute kidney injuries. The offending drug or agent should be defined swiftly and must be stopped. In some situations, the culprit agent may not be obvious. Energy drink consumption has reportedly increased in recent years, with ads claiming that energy drinks strengthen physical and mental performance. However, when consumed in uncontrolled amounts, they may have negative effects on health. An energy drink-induced acute kidney injury is reported in this case presentation. There have been only four similar cases in the literature.

**Case presentation:**

A 21-year-old male patient of Turkish origin applied to the emergency department with nausea, vomiting, and malaise. He was admitted, because the laboratory values revealed that he had stage 3 acute kidney injury. He did not have any medical or surgical histories. He did not use any drugs, but he stated that he has consumed 2 L of an energy drink product per day for the past month. Differential diagnosis work-up pointed to an energy drink-induced acute kidney injury. His serum creatinine increased to a level as high as 10.32 mg/dL, but he did not need any renal replacement therapy. Creatinine levels normalized in about 2 weeks after withholding energy drinks.

**Conclusion:**

Depending on their content and consumption amount, energy drinks may cause acute kidney injury. There have been four previous cases in the literature and they were reviewed for a comparison with this case.

## Background

Acute kidney injury (AKI) is an important health problem with an increasing incidence [1]. The etiology should be defined and relevant treatment should be started swiftly [2]. Etiologies of AKI may be different for hospital-acquired (HA) and community-acquired (CA) AKIs. Infections, volume loss, glomerular diseases, urinary tract obstruction, and nephrotoxins are leading causes of CA-AKIs while surgeries, sepsis, and drug toxicity are responsible for HA-AKIs [3]. Prior kidney diseases or other risk factors including diabetes or heart failure may ease the occurrence of AKI [4]. Differential diagnosis algorithm should be rigorously applied for all patients with AKI since glomerular or interstitial diseases carry a high burden of morbidity. Nephrotoxins should also be questioned, as timely elimination of the offending agent is necessary for recovery [5]. The toxic agent may not always be apparent. Herein, I report a case of AKI in a 21-year-old healthy male patient, who has been drinking two L of “energy drink” each day to prepare for a running race. To my knowledge, there have been four previous cases reporting AKI with high consumption of energy drinks. The presented case is given in comparison with previous reports.

## Case presentation

A 21-year-old male patient of Turkish origin applied to the emergency department with nausea, vomiting, and malaise that started the previous day. He did not have any previous medical, surgical, or interventional histories. There was no history of kidney diseases or any other diseases in his family either. He was a nonsmoker and seemed to be a fit healthy young man. He stated that he was not a substance abuser and has never used any illicit drugs. His weight and height were 72 kg and 1.74 m with a body mass index of 23.7 kg/m^2^. Upon physical examination, he was euvolemic, lungs were clear to auscultations, heart sounds were normal, and there was no pretibial edema. Blood pressure was 115/75 mmHg and he had sinus rhythm with a heart rate of 59 beats per minute. Laboratory results in the emergency unit were as follows; creatinine: 5.31 mg/dL, urea: 93 mg/dL, LDH: 299 U/L, calcium: 9.9 mg/dL, sodium: 141 mmol/L, potassium: 3.8 mmol/L, phosphorus: 7.7 mg/dL, ALT: 18 U/L, AST: 14 U/L, total bilirubin: 0.8 mg/dL, direct bilirubin: 0.27 mg/dL, creatine kinase: 557 IU/L, WBC: 11.69 × 10^3^/μL, hemoglobin: 15.8 g/dL, platelets: 259 × 10^3^/μL. There was no acidosis in blood gas analysis with a HCO_3_ level of 23.6 mmol/L. Urinary ultrasound was completely normal. In initial inquiry, he did not report use of any nephrotoxic drugs or agents. He had stated that he had been preparing for a running race for more than a month and that to be better prepared, he had been consuming two L of “energy drink” on a daily basis, which he bought from a local market. Toxicological tests that checked for alcohol, amphetamine, cannabinoids, 3,4-methylenedioxymethamphetamine (MDMA; ecstasy), opiate morphine, and cocaine metabolites were negative. The patient was admitted to the nephrology clinic and subsequently stopped drinking the energy drink. His urine volume in the first day of hospital admission was 100 mL. Creatinine level increased to 7.05 mg/dL, 9.19 mg/dL, and 10.32 mg/dL on the 2nd, 3rd and 4th days, respectively. Urinalysis was free of hematuria or proteinuria and urine density was 1.005. Urine culture that was obtained upon admission resulted as sterile. Urine volume was 300 mL in the 2nd day and 800 mL in the 3rd day. Electrolytes were in the normal range and the patient did not have hypervolemia or acidosis in the follow-up. Thus, he did not need hemodialysis. Tests for hepatitis B, hepatitis C, and human immunodeficiency virus (HIV) were negative. Serologic tests for antinuclear antibodies (ANA), anti–double-stranded DNA antibodies (anti-ds DNA), and antineutrophil cytoplasmic antibodies (ANCA) profile were also negative. Urine volume increased to 1250 mL in the 4th day and creatinine decreased for the first time to a level of 9.55 mg/dL the following day. Recovery phase, with prominent polyuria, started in the 5th day. Creatinine levels gradually decreased and normalized on 16th day of the admission. The course of urea, creatinine, and urine volume levels can be seen in Table 1. During the 2-year follow-up period, renal functions remained normal.

**Table 1:** The changes in urea, creatinine and urine volume during the follow-up

	Urea (mg/dL)	Creatinine (mg/dL)	Urine Volume (mL)	CRP (mg/L)
Admission	93	5.31	100	37.38
Day 2	107	7.05	300	33.97
Day 3	124	9.19	800	39.56
Day 4	144	10.32	1250	44.36
Day 5	136	9.55	3350	36.97
Day 6	133	8.29	4500	–
Day 7	127	6.33	5500	–
Day 8	103	4.53	7000	–
Day 9	87	3.25	6000	–
Day 10	54	2.33	6000	–
Day 11	55	1.90	5000	–
Day 12	45	1.67	4500	6.93
Day 16	46	1.20	4000	–

## Discussion and conclusion

AKI in this case was a stage 3 AKI (more than threefold increase in basal creatinine) according to Kidney Disease Improving Global Outcomes (KDIGO) criteria [2]. Nephrotoxins may be responsible for CA-AKIs, which are more likely to progress to stage 3 than HA-AKIs [4]. The toxic drug or agent may not always be obvious. EDs were reported to cause AKIs in a couple of previous reports. Case reports included patients in their teenage years to those in their 60s [6–9]. All, but one of the cases, consumed equal to or more than 2 L of ED a day for many consecutive days. In one of the cases, 3 L of ED was consumed at once but with a high dose of alcohol [6]. ED was consumed with alcohol in two of the reported cases and one other reported case was a previous alcohol addict. Kidney biopsy was not performed in any of the reported cases. We also did not perform a biopsy as oliguria resolved after 3 days. It would have been beneficial to reveal the renal pathology with a biopsy; however, this would be an unnecessary intervention, as there is no indication for kidney biopsy in resolving AKI cases. In differential diagnosis, glomerular diseases were not likely as there was no proteinuria or hematuria. In addition, autoimmune markers and serologic tests were all negative. Other differential diagnosis of AKI in a young athlete include prerenal AKI due to dehydration, use of nonsteroidal antiinflammatory drugs (NSAIDs), and exertional rhabdomyolysis. The patient was euvolemic at presentation and thus dehydration was not a possible etiology. Furthermore, he did not report use of any drugs including NSAIDs. Exertional rhabdomyolysis is also unlikely, as creatine kinase was not that high. The clinical picture of this patient and inferences from previous literature point to acute tubular necrosis (ATN) as the most probable pathology. During the 2-year follow-up of this case, AKI did not recur or progress to chronic kidney disease. Follow-up data were limited in other reported cases; however, AKI was generally fully reversible. In one of the cases, although remaining stable, renal functions did not fully recover during the 10 months of follow-up [7].

Our patient consumed an energy drink that contained 150 mg/L of caffeine, 800 mg/L of taurine, 100 mg/L of inositol, 20 mg/L of glucuronolactone, 80 mg/L of niacin, and vitamins B2 (6 mg/L), B6 (20 mg/L), and B12 (20 μg/L). Sodium benzoate and potassium sorbate were added as preservatives to the product. The exact reason for AKI after ED consumption is not very obvious. Previous case reports underlined taurine or an interplay between taurin and caffeine for the toxic effect. In an experimental study on rats, use of a commercially available ED as well as an in-house combination of caffeine and taurine resulted in hyaline degeneration and hemorrhage in the kidneys [10]. In the same study, urinary levels of *N*-acetyl-beta-d-glucosaminidase (NAG), as a marker of tubular damage, increased in rats that were exposed to ED and alcohol combination. This underlines the potentiated nephrotoxic effect of EDs when consumed with alcohol. In contrary, there are also experimental models pointing to the reno-protective effects of taurine with its antioxidant potential [11]. We were not able to check blood caffeine or taurine levels in our patient owing to the unavailability of these tests in our institution. Further studies may be required to define the possible relations between taurine, caffeine, and kidney tissue. However, EDs are sugary drinks that include glucose and fructose. It was previously shown that, consumption of such drinks during or following exercise may also provoke AKI by resulting in decreased renal perfusion due to mild dehydration and increased circulatory vasopressin [12]. The possible toxic effects of preservatives such sodium benzoate should also be noted [13], although their dose could not be found for the product consumed by the patient.

The most important first step to treat ED-induced AKI is withholding it. Fluid and electrolyte therapy should be planned as in other forms of AKI. Renal replacement therapy may be needed, depending on the electrolytes, acid–base status, and volume load of the patient. While hemodialysis was not needed in this case, it was needed in two of the previously reported cases. Given that the most likely pathology is acute tubular necrosis, plateaus for creatinine level is expected to occur in a week. Recovery has occurred in about 2 weeks’ time in all reported cases, as also expected in the course of ATN (Table 2).

**Table 2:** Previous cases reporting energy drink induced acute kidney injury for a comparison with the present case

Reference No.	Age	Sex	Consumed amount and time	Taurine content	Caffeine content	Concomitant agents	Hemodialysis requirement	Other systems involvement	Recovery after discontinuation
[6]	17	Male	3 L (once)	1500 mg/L	260 mg/L	Alcohol (1 L of vodka)	Yes	None	10 days
[7]	40	Male	2 L a day (for weeks)	800 mg/L	150 mg/L	Ibuprofen, Lisinopril	No	None	3 days
[8]	30 s	Male	3 L a day (for days)	Not given	320 mg/L	Alcohol	Yes	Psychosis	17 days
[9]	62	Female	2.5 L a day (for weeks)	4000 mg/L	320 mg/L	None	No	Hepatitis	10 days
Present Case	21	Male	2 L a day (for a month)	800 mg/L	150 mg/L	None	No	None	16 days

ED-related toxic effects might be seen in other organ systems; some examples are the central nervous system [8], liver [9], and heart [12]. Although cardiomyopathy [14] and increased blood pressure [15] may have deleterious effects in the long term, many of the organ systems involvement are reversible after withholding the ED. Physical examination or laboratory tests did not point out any other systems involvement in this case. Both healthcare professionals and the public should be aware that energy drinks can have harmful effects and that these effects increase with the amount consumed. In conclusion, depending on their content and consumption amount, EDs may cause AKIs. As it is reported that the consumption of EDs has substantially increased in the recent years [16], they should be questioned, especially in younger patients presenting with AKIs.

## Data Availability

The data of this case report is available from the corresponding author upon a reasonable request.

## Abbreviations

AKI: Acute kidney injury
ATN: Acute tubular necrosis
CA-AKI: Community-acquired acute kidney injury
ED: Energy drink
HA-AKI: Hospital-acquired acute kidney injury
KDIGO: Kidney disease improving global outcomes
